# Limitations and Challenges in the Stability of Cysteamine Eye Drop Compounded Formulations

**DOI:** 10.3390/ph15010002

**Published:** 2021-12-21

**Authors:** Cristina Martín-Sabroso, Mario Alonso-González, Ana Fernández-Carballido, Juan Aparicio-Blanco, Damián Córdoba-Díaz, Federico Navarro-García, Manuel Córdoba-Díaz, Ana I. Torres-Suárez

**Affiliations:** 1 Department of Pharmaceutics and Food Technology, Complutense University of Madrid, 28040 Madrid, Spain; crmartin@ucm.es (C.M.-S.); marioalonsogonzalez@ucm.es (M.A.-G.); afernand@farm.ucm.es (A.F.-C.); juan.aparicio.blanco@ucm.es (J.A.-B.); damianco@ucm.es (D.C.-D.); mcordoba@ucm.es (M.C.-D.); 2 Institute of Industrial Pharmacy, Complutense University of Madrid, 28040 Madrid, Spain; 3 Microbiology and Parasitology Department, Complutense University of Madrid, 28040 Madrid, Spain; fnavarro@farm.ucm.es

**Keywords:** cysteamine, ocular cystinosis, stability, eye drop formulations

## Abstract

Accumulation of cystine crystals in the cornea of patients suffering from cystinosis is considered pathognomonic and can lead to severe ocular complications. Cysteamine eye drop compounded formulations, commonly prepared by hospital pharmacy services, are meant to diminish the build-up of corneal cystine crystals. The objective of this work was to analyze whether the shelf life proposed for six formulations prepared following different protocols used in hospital pharmacies is adequate to guarantee the quality and efficacy of cysteamine eye drops. The long-term and in-use stabilities of these preparations were studied using different parameters: content of cysteamine and its main degradation product cystamine; appearance, color and odor; pH and viscosity; and microbiological analysis. The results obtained show that degradation of cysteamine was between 20% and 50% after one month of storage in the long-term stability study and between 35% and 60% in the in-use study. These data confirm that cysteamine is a very unstable molecule in aqueous solution, the presence of oxygen being the main degradation factor. Saturation with nitrogen gas of the solutions offers a means of reducing cysteamine degradation. Overall, all the formulae studied presented high instability at the end of their shelf life, suggesting that their clinical efficacy might be dramatically compromised.

## 1. Introduction

Cystinosis is a rare autosomal recessive disorder that affects the lysosomal storage of the amino acid cystine. Its prevalence is estimated at around 1/100,000–1/200,000 among live births. This systemic disease is characterized by mutations in the CTNS gene located on chromosome 17p13 that lead to the defective production of the ubiquitous lysosomal transmembrane cystinosin, which is responsible for the transport of cystine out of the lysosome [1]. As a result, cystine builds up in lysosomes as crystals, which in turn leads to the progressive impairment and dysfunction of multiple organs and tissues, including the kidneys and the eyes [2,3,4].

The tear film is the main barrier of the eye followed by the cornea. In this structure three membranes are distinguished: the epithelium, stroma and endothelium. The outermost layer is the corneal epithelium, lipophilic in nature; the stroma is the intermediate layer, with a highly hydrophilic layer, and makes up of 90% of the cornea; and the corneal endothelium, with a single layer, is responsible for maintaining normal corneal hydration [5]. Ocular manifestations are typical features of all forms of cystinosis (infantile nephropathic, juvenile and ocular non-nephropathic) [6,7]. Cystine crystals are present in all ocular structures including the cornea and are responsible for photophobia due to light reflections (glare sensitivity, blepharospasm) and corneal erosions [8,9], and can lead to several complications, including keratopathy, retinopathy and ultimately blindness [9].

Current treatments rely on the administration of cysteamine (mercaptamine hydrochloride). This aminothiol lowers intra-lysosomal cystine levels by binding to cystine to create cysteine and cysteine–cysteamine mixed disulfide, which does not rely on the cystinosin export system, and provides an alternative means of cystine clearance in lysosomes. Oral cysteamine has been proved to considerably delay the progression of the disease and increase life expectancies. However, it has no effect on the accumulation of cystine in the cornea due to its lack of vascularization [10,11].

To overcome this problem, cysteamine must be administered by ocular route to reduce the number of crystals present in the cornea. However, cysteamine is very easily oxidized, giving rise to cystamine as the main degradation product [12]. The formation of cystamine is pH dependent, being more pronounced in alkaline solutions, and increases with increasing temperature [13]. This compound lacks therapeutic activity, which can lead to treatment failure with serious consequences for patients [14].

Currently, there are very few commercial cysteamine eye drops. Specifically the European Medicines Agency (EMA) has only approved one medicine, Cystadrops^®^, which was designated an orphan drug in 2017 [15]. In the case of the Food and Drug Administration (FDA), there are two medicines approved, Cystaran^®^ and Cystadrops^®^, which received marketing authorization in 2012 and 2020, respectively [16,17]. However, these formulations are not currently available in many countries [18,19,20]. In the absence of a marketed medicinal product, cysteamine eye drops have to be prepared in hospital pharmacies, from different excipients and protocols, and in many cases their shelf life is not supported by rigorous stability studies. Indeed, due to the rapid oxidation of cysteamine, there is a risk that these solutions are unable to prevent corneal cystine deposits and ocular complications [21,22,23,24,25].

Stability studies to be carried out on the different cysteamine eye drop compounded formulations should include not only a long-term stability study covering their proposed shelf life but also an in-use stability test reproducing the daily opening conditions of the containers.

Therefore, the main objective of this work was to determine if the shelf life proposed for six formulations of cysteamine eye drops prepared in hospital pharmacies following different protocols is adequate to guarantee their quality and efficacy. For this, their chemical (cysteamine and cystamine content), physical (appearance, color, odor, pH and viscosity) and microbiological stability were evaluated by long-term and an in-use stability studies. The influence of excipients, the manufacturing process and storage conditions on the stability of the formulations were analyzed.

## 2. Materials and Methods

### 2.1. Materials

Cysteamine hydrochloride BioXtra, cysteamine purity 98%, cystamine impurity standard, orthophosphoric acid 85% and acid 1-heptanosulphonic, sodium salt were obtained from Sigma-Aldrich (St. Louis, MO, USA). Hyaluronic acid, with a molecular weight of 10^6^ Da and a purity of 97%, was purchased from Acofarma (Madrid, Spain). Ethylenediaminetetraacetic acid disodium salt (EDTA) was obtained from Fagron (Barcelona, Spain). HPLC grade acetonitrile was purchased from Thermo Fisher Scientific (Frederick, MA, USA). Balanced salt solution (BSS) was purchased from Alcon (Geneva, Switzerland). Hydrochloric acid 1M was obtained from Carlo Erba (Barcelona, Spain). Ultrapure water MilliQ, Merck Millipore (Madrid, Spain) was used. Class I 15 mL amber glass dropper bottles were used as primary packaging (Fagron, Barcelona, Spain).

### 2.2. Preparation of Cysteamine Hydrochloride Sterile Aqueous Solutions

Different cysteamine solutions at 0.55% were prepared from cysteamine hydrochloride following different protocols used at the hospital pharmacy level. Formulae I–IV correspond to a cysteamine and hyaluronic acid hydrogel developed by Fernandez-Ferreiro et al. [26] (Table 1). In these formulations BSS was used as the aqueous vehicle, in which the drug and hyaluronic acid were dissolved. To evaluate the effect of the incorporation of a chelating agent on the stability of the formulations, EDTA was incorporated in FI and FII solutions, at a concentration of 0.01%, prior to hyaluronic acid addition. Finally, all solutions were filtered under vacuum using a 0.22-micron membrane filter (Stericup^®^ Merck Millipore ExpressTM Plus 0.22 μm) and dosed in amber glass dropper bottles. Before closing, FI and FIII formulations were saturated with nitrogen gas to evaluate the role of oxygen in the stability of the cysteamine.

Formulae V and VI have the simplest cysteamine composition: physiological saline solution was used as an aqueous vehicle, in which cysteamine and the chelating agent EDTA were incorporated. Once both components were dissolved, the solution was filtered through a 0.22 µm syringe filter, and sterile amber glass dropper bottles were filled with it [27]. Both formulations were identical and only differed with respect to storage conditions in order to evaluate the influence of environmental temperature on cysteamine solution stability (Table 1).

In all cases, the entire manufacturing and packaging process took place in aseptic conditions in laminar flow hoods following the official standard manufacturing protocol for sterile formulae. All the materials used and the bottles were previously sterilized.

### 2.3. Long-Term Stability Study

Once the formulations were prepared, the samples of formulae I–V were stored in a refrigerator at a controlled temperature of 5 °C ± 3 °C, whereas FVI samples were stored in a climate chamber at room temperature (25 °C ± 0.5 °C) [28].

The duration of this stability study was 1 month. This is the commonly established shelf life for the cysteamine eye drops elaborated in hospital pharmacies.

At each sampling time (0, 3, 7, 15, 21 and 30 days), two bottles of each formula were withdrawn and physical and chemical properties were determined. At the beginning and the end of the study, three additional bottles were withdrawn for microbiological testing. Samples were tempered at 25 °C for 20 min before analysis.

### 2.4. In-Use Stability Study

During this study, the conditions of formulation usage were simulated: opening the vial and removing two drops of each solution four times a day for 7 days [20,22,23]. This was the in-use shelf life that was generally proposed in the literature for these cysteamine eye drops elaborated in hospitals.

At each sampling time (0, 3 and 7 days), two vials of each formula were withdrawn to determine their physical properties and chemical composition. Additionally, on the day of preparation of the formulations and the last day of the study, three extra vials were withdrawn for microbiological testing. All samples were tempered at 25 °C for 20 min before analysis.

### 2.5. Physical Stability

#### 2.5.1. Appearance, Color and Odor

After opening the bottle and transferring the content to a transparent glass container, the organoleptic properties of each aqueous sterile solution were determined.

#### 2.5.2. pH and Viscosity

pH (PH meter GLP22, Crison) was determined with a resolution of 0.01 units. Regarding viscosity, an Ostwald viscosimeter (Comecta serie 100) was used, and data were presented as relative viscosity compared to water, with a value of 1.

Osmolarity was not evaluated because the different formulae used in this study did not have the osmolarity adjusted to physiological osmolarity. As is usual in ocular preparations, all the formulae have been made from an isotonic vehicle (in this case, saline or BSS) and are therefore slightly hypertonic.

### 2.6. Chemical Stability

The content of cysteamine and its main degradation product (cystamine) in the aqueous sterile solutions were determined by high-performance liquid chromatography (HPLC). An Agilent 1200 Infinity Quaternary HPLC system with a variable UV wavelength detector and autosampler was utilized. The mobile phase was a mixture of 0.5% aqueous sodium heptane sulfonate solution adjusted at pH 2.5 with phosphoric acid 85%: acetonitrile, in gradient mode, at a flow rate of 1.0 mL/min (Table 2). A reversed-phase C18 column (3.5 μm, 15 × 0.46 cm, Scharlab) kept at 30 °C during analysis was used. The wavelength used was 210 nm. Under these conditions, cysteamine elutes at a retention time of 3.65 min and cystamine at 6.68 min.

The analytical method was previously validated, being linear (r = 0.997) in a range of 130–30% of the labelled cysteamine concentration, accurate (individual recoveries were within a 96.6–101.0% interval, and the mean recovery was equal to 98.5%) and precise (RSD_repeatability_ = 0.91% and RSD_intermediate precision_ = 1.78%). The reporting threshold (0.05% of labelled cysteamine eye drops concentration) [29] was validated as the LOQ, being linear in a range of 0.05–10%, accurate (mean recovery of 94.9%) and precise (RSD_repeatability_ = 6.7% and RSD_intermediate precision_ = 7.3%). Analytical solutions were stable for at least 10 h at 25 °C in vial for injection.

For the quantification of the drug and its degradation product throughout the stability studies, two standard solutions of cysteamine hydrochloride, 550 µg/mL for cysteamine quantification and 2.75 µg/mL for cystamine quantification (reporting threshold), were prepared. The response factor in case of cystamine degradation was 0.87.

Test samples of formulae V and VI were diluted 1:10 and were analyzed by HPLC, with a 20 µL injection volume. Formulae I–IV, more viscous due to hyaluronic acid, were diluted 1:20 and analyzed by HPLC setting a 40 µL injection volume.

In all cases a 50:50 mixture of ultrapure water (Milli-Q) and 0.1 M aqueous HCl solution was used as diluent.

### 2.7. Microbiological Stability

The sterility of the formulations was tested for compliance. The studies were selected to cover the possible contamination of the samples with both aerobic and anaerobic microorganisms as well as possible fungal contaminants [30]. Briefly, blood agar (BA), fluid thyoglycollate medium (TG) and Sabouraud agar (SA) were inoculated with samples of at least 3 different containers of each formulation and incubated at 37 °C for 48 h (BA) or 14 days (TG) and 28 °C for 14 days (SA). For BA and SA, the membrane filtration test was performed by diluting 1 mL of each formulation with 9 mL of saline solution and vacuum filtering through a 0.22 micron membrane under aseptic conditions. In order to avoid possible antimicrobial properties of the product in the assay, filters were washed 3 times with saline solution and finally disposed on plates of the corresponding media. In the case of the TG medium, direct inoculation of 1 mL of each formulation in 10 mL of medium was achieved. Inoculated plates and tubes were incubated as indicated before. A growth promotion test was performed similarly by inoculating 100 CFU per BA plate or TG media with *Staphylococcus aureus* ATCC 6538 (bacterial tests) or SA plate with *Candida albicans* ATCC 10231 (fungal tests). Intrinsic activity was measured in TG media by inoculating *S. aureus* ATCC 6538 in TG media. Non-inoculated media, as well as controls of dilution solutions, were also tested using the three media as indicated before. In all cases, microbial growth was determined by visual inspection after the corresponding incubation periods.

## 3. Results

### 3.1. Preparation and Characterization of Cysteamine Hydrochloride Sterile Aqueous Solutions

During the preparation and packaging of the formulations, aseptic conditions were maintained by laminar flow hoods. All the formulations showed a clear and transparent appearance, highlighting that the formulations containing hyaluronic acid required a more intense and extended stirring. Later, due to the higher viscosity of these formulations, their filtration was slower.

Significant differences were detected in the initial pH of the different formulae. The formulae FI–FIV, with hyaluronic acid and BSS as vehicle, presented pH values above 6, probably due to the pH buffering power of the vehicle. In contrast, in the FV and FVI formulae, without hyaluronic acid and with a simple solution of sodium chloride in water as vehicle, the initial pH value was around 5.5. Regarding the formulae made with BSS, those that contained EDTA (FI and FII) showed pH values 0.2–0.3 units higher than the formulae that did not contain EDTA (FIII and FIV).

As expected, the FV and FVI formulations had a viscosity equal to water. On the contrary, the formulations FI, FII, FIII and FIV showed, freshly prepared, a viscosity much higher than water, due to the presence of hyaluronic acid. This viscosity was slightly higher in formulae that did not contain EDTA (FIII and FIV).

Once the formulations were characterized, they were packed in aliquots of 10 mL in amber glass dropper bottles and, prior to closing the containers, the saturation with nitrogen gas of formulations FI and FIII was carried out. Finally, the samples were stored in the refrigerator or at room temperature for the long-term and in-use stability studies.

### 3.2. Long-Term Stability Study

#### 3.2.1. Appearance, Color and Odor

No color changes or turbidity was observed in any of the samples throughout the long-term stability study.

Regarding odor, after a certain time an unpleasant odor of hydrogen sulfide was detected in the samples, which became more intense with increasing storage time. Specifically, in formula FVI this odor was already detected in some of the samples after 3 days of storage; in formulae FII, FIV and FV, after 7 days; in formula FI, after 15 days; and in formula FIII, after 21 days.

Therefore, this hydrogen sulfide odor appeared later in samples saturated with N2 (FI and FIII), and especially in the formulation without EDTA (FIII).

#### 3.2.2. pH and Viscosity

**Figure 1:**
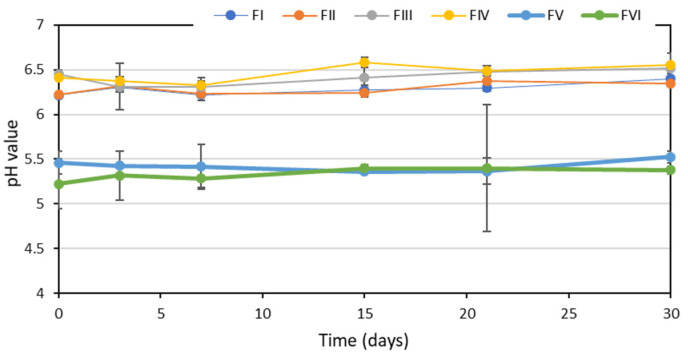
Evolution of the pH values of the different formulae during the long-term stability study.

Regarding the pH, fluctuations in the pH values of formulations FI, FII, FIII and FIV throughout the storage time were very small, not exceeding 0.4 units. This was probably due to some buffering effect of the pH exerted by the balanced salt solution used as the vehicle in these formulae. In formulae FV and FVI, with saline as the vehicle, despite the fact that the mean pH values show almost no difference, when analyzing the individual data, the fluctuations in the pH values were somewhat greater, especially in formula FVI, in which differences of up to one pH unit were detected (Figure 1). These fluctuations in pH could not be associated with the appearance of a degradation product, since these products do not modify pH.

**Figure 2:**
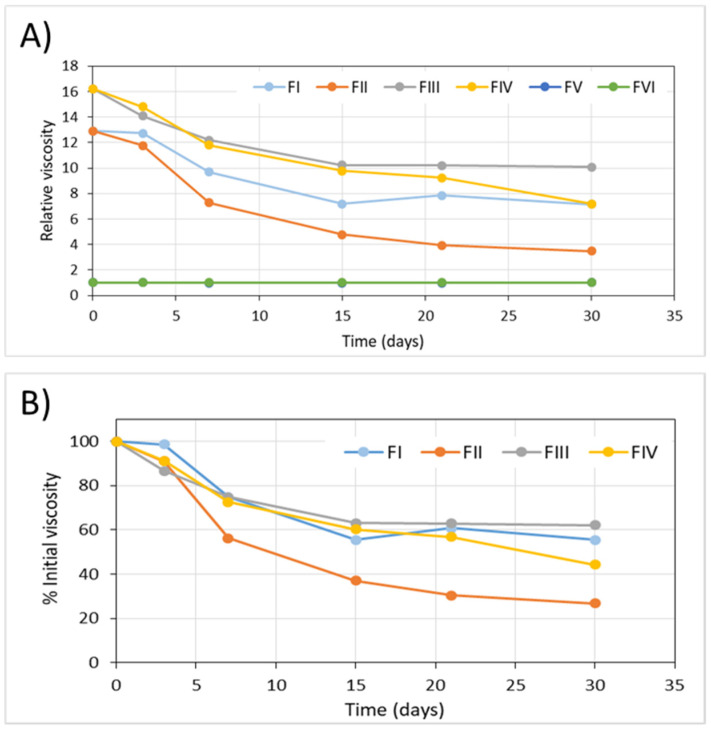
Evolution of the viscosity values of the formulae throughout the long-term stability study. (**A**) Relative viscosity (water = 1). (**B**) Variation in relative viscosity with respect to the initial value (%).

Regarding the viscosity, for the FV and FVI formulations, their viscosity did not vary throughout the long-term stability study and their patterns appear superimposed in Figure 2a. However, the formulations with hyaluronic acid showed a marked decrease in viscosity over time, so that after one month of storage at 5 °C values ranging between 62% (FIII) and 27% (FII) of the initial value were obtained (Figure 2b).

#### 3.2.3. Chemical Stability

The cysteamine and cystamine content in the samples were evaluated at all the established sampling times, detecting in all the formulae a degradation of more than 20% in the active ingredient after one month of storage.

**Figure 3:**
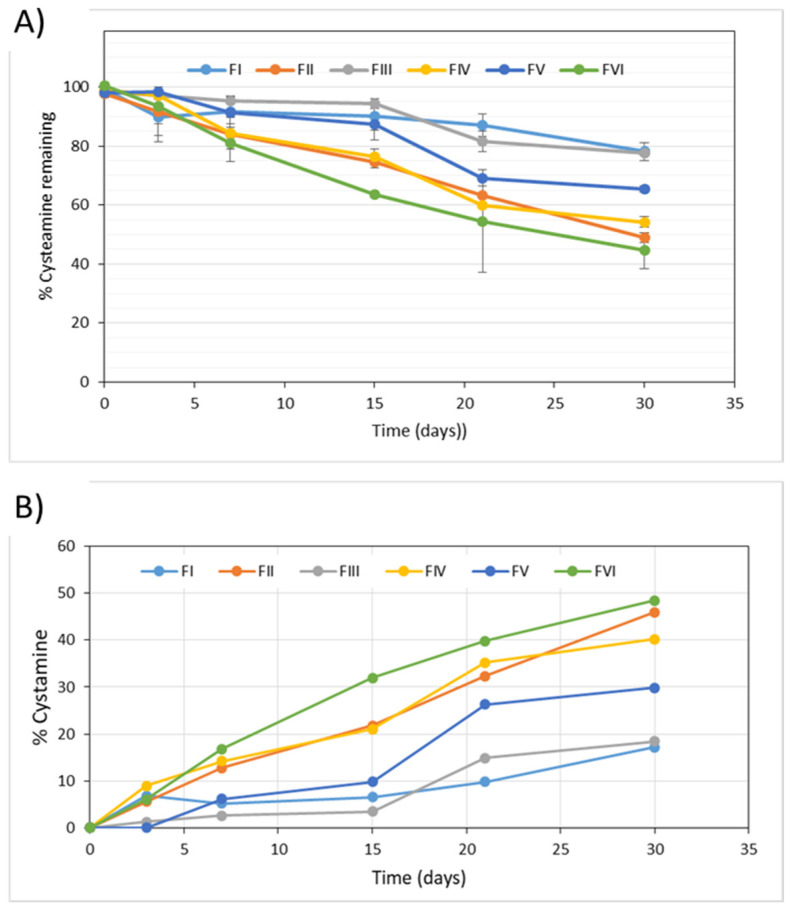
(**A**) Percentage of remaining cysteamine during the long-term stability study. (**B**) Evolution of cystamine levels in the different formulae throughout the long-term stability study.

The most unstable formula was FVI, stored at room temperature, in which the amount of drug remaining after 30 days was less than 50%. The most stable formulae were FI and FIII, stored in the refrigerator, and characterized because they were saturated with nitrogen gas. In these formulae the percentage of drug remaining after one month of storage was around 80% (Figure 3).

Although the differences were not significant, higher correlations were obtained assuming zero-order degradation kinetics. The degradation rate constants calculated for each formulation based on long-term stability data are listed in Table 3. The comparison of the formulae with an identical composition but with or without nitrogen saturation (FI vs. FII and FIII vs. FIV) indicated that nitrogen saturation reduced the oxidation of the drug in the formulation, since the degradation rate constant values were multiplied by three and by two in the absence of nitrogen, so that in the formulae not saturated with nitrogen the percentage of degradation of the drug was double in the same period of time. On the other hand, the comparison of the formulae with EDTA (FI and FII) or without EDTA (FIII and FIV) in their composition did not show differences between the two formulations, regardless of whether the formula was saturated with nitrogen or not (differences in degradation rate constants between formulae were not statistically significant). When comparing all the formulae without nitrogen saturation, the greater stability of the FV formula was striking, with the statistically lowest value of degradation rate constant, and with remaining cysteamine values of 65% after one month of storage compared to values slightly higher than 50% for the FII and FIV formulations. The FV formulation had a much simpler composition than FII and FIV, since, although it contained EDTA, it did not contain hyaluronic acid, and the vehicle was a simple sodium chloride solution instead of BSS for ophthalmic use.

Finally, when comparing the FV and FVI formulations, with the same composition, the effect of the storage temperature on the stability of cysteamine was evident, with a significantly higher degradation rate constant and 20% more degraded cysteamine when the samples were stored at room temperature (25 ± 0.5 °C in formula VI) instead of in the refrigerator (5 ± 3 °C in formula V). The ratio of the degradation rate constants at 25 °C and 5 °C is 1.58.

After one month of storage, the drug content in the formulae stored in the refrigerator ranged between 78% (FI and FIII) and 50% (FII and FIV) of the labeled content.

The values of cystamine, the main degradation product of cysteamine, detected in the six formulae evaluated in the long-term stability study, are represented in Figure 3b.

After 1 month of storage, cystamine was detected at a level greater than 15% in all formulae. The highest percentage of cystamine (about 50%) was obtained with the FVI formula, stored at room temperature, while the lowest percentage of cystamine was obtained in formulae FI and FIII, stored in the refrigerator and saturated with N2. In these formulae the cystamine level after one month of storage was around 18%.

The comparison of the formulae with the same composition but with or without nitrogen saturation (FI vs. FII and FIII vs. FIV) showed that saturation with nitrogen reduced the generation of the main degradation product. In the formulae without nitrogen, cystamine levels higher than 40% were reached after one month of storage, while in the formulae with nitrogen the percentage of cystamine did not reach 20% in the same period of time.

As with cysteamine, no significant differences were detected in the cystamine levels between the formulae with EDTA (FI and FII) or without EDTA (FIII and FIV) in their composition.

When comparing all the formulae without nitrogen saturation, it was confirmed that the formula FV, with a simpler composition, presented lower values of the degradation product—30%—compared to values close to 45% for the FII and FIV formulations.

The effect of the storage temperature was appreciated when comparing the formulations FV and FVI, with the same composition: the formula FV presented, after one month stored in the refrigerator, 30% of the cystamine degradation product, while in formula VI, conserved at 25 °C, this value was 48%.

After one month of storage of the formulae FI–FIV, the content of the main degradation product, cystamine, ranged between 17% (FI) and 48% (FII).

**Figure 4:**
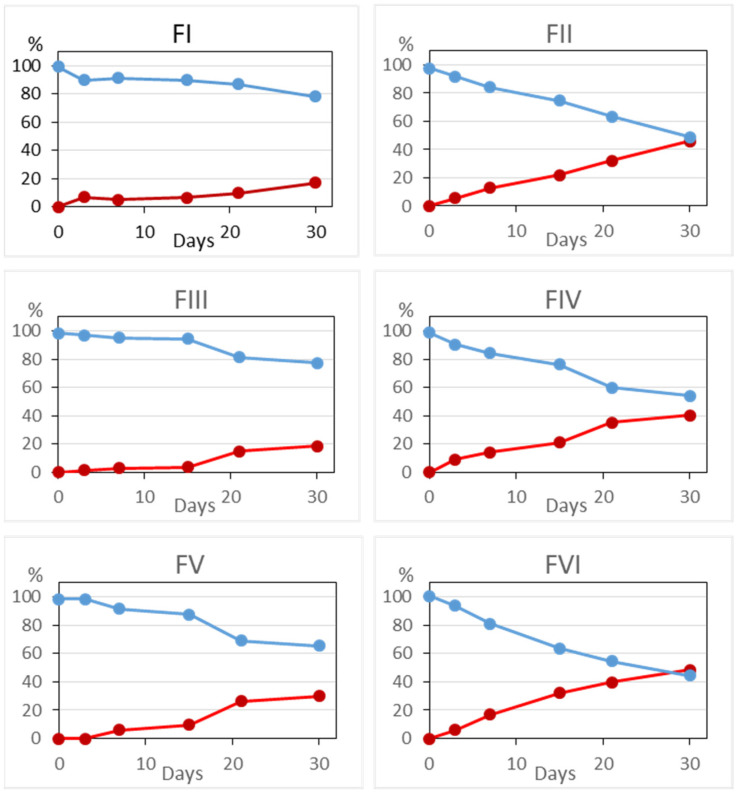
Evolution of cysteamine 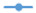 and cystamine 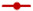 levels in the six formulae studied.

In all cases, there was a clear parallelism between the increase in the levels of the degradation product cystamine in all the formulae and the decrease in the content of the drug cysteamine, as can be seen in Figure 4.

#### 3.2.4. Microbiological Stability

All formulations were stable from a microbiological point of view along long-term stability conditions since no contamination was detected in any of the evaluated media (blood agar, thioglycolate and Sabouraud agar).

### 3.3. In-Use Stability Study

The samples destined for in-use stability studies were handled according to the guidelines established for the administration of these formulations. Thus, every 6 h the samples were opened and two drops instilled from each container.

#### 3.3.1. Appearance, Color and Odor

No coloration or turbidity was observed in any of the samples throughout the in-use stability study, except in the case of one of the samples of the formulation FI, in which particles in suspension were observed after 7 days.

Regarding odor, three days after preparation an unpleasant odor of hydrogen sulfide was detected in most of the samples, which became more intense as in-use time increased. In the formulae FV and FVI, this odor was already detected after three days in all samples, and was more intense in the case of the FVI formula.

#### 3.3.2. pH and Viscosity

**Figure 5:**
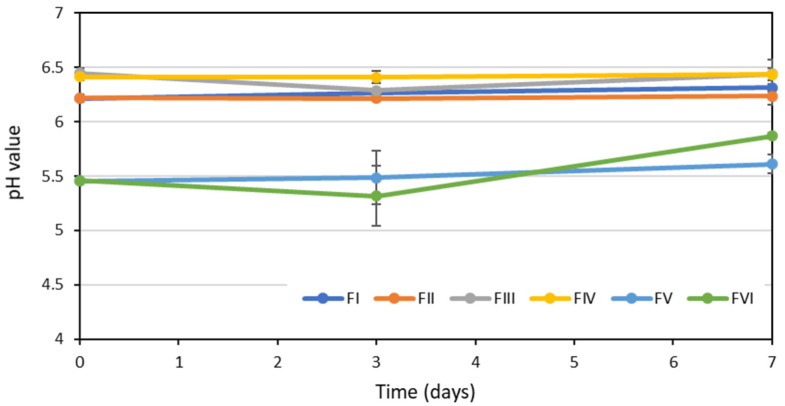
Evolution of the pH values of the different formulae throughout the in-use stability study.

As already observed in the long-term stability study, the fluctuations in the pH values of the formulations FI–FIV throughout the in-use period were very small (less than 0.2 units) due to the buffering effect of the balanced saline solution used as a vehicle (Figure 5). In the formulae FV and FVI, with saline as a vehicle, greater fluctuations in the pH values were observed again, especially in the formula FVI; analyzing the individual data, these were higher than 0.7 units.

A slight increase in pH in formulae FI, FV and FVI was detected as the in-use time increased. This increase was not detected in formulae FII, FIII and FIV.

**Figure 6:**
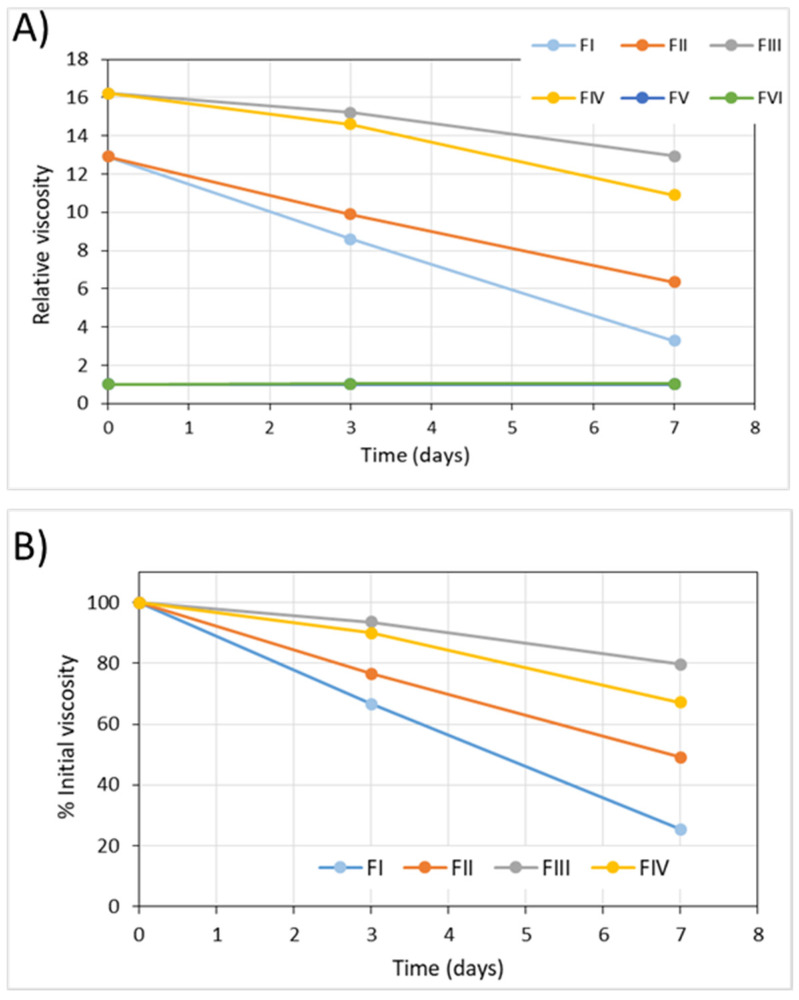
Evolution of the viscosity values of the formulae in the in-use stability study. (**A**) relative viscosity (water = 1). (**B**) Variation in relative viscosity with respect to the initial value (%).

As in the long-term stability study, the viscosity of the formulations FV and FVI did not vary throughout the in-use stability study (Figure 6).

On the contrary, in the FI, FII, FIII and FIV formulations that presented, freshly prepared, a viscosity much higher than water due to the presence of hyaluronic acid, a marked decrease in viscosity with time was observed, just as was found in the long-term stability study. It was in the formulae containing EDTA that a greater change in viscosity was observed. In the case of FI, after 7 days of use, viscosity was only 25% of the initial value. In this in-use stability study, no minor decrease in viscosity values was observed in formulae saturated with nitrogen because after opening the container the effect of the gas on the formulation ceased.

#### 3.3.3. Chemical Stability

**Figure 7:**
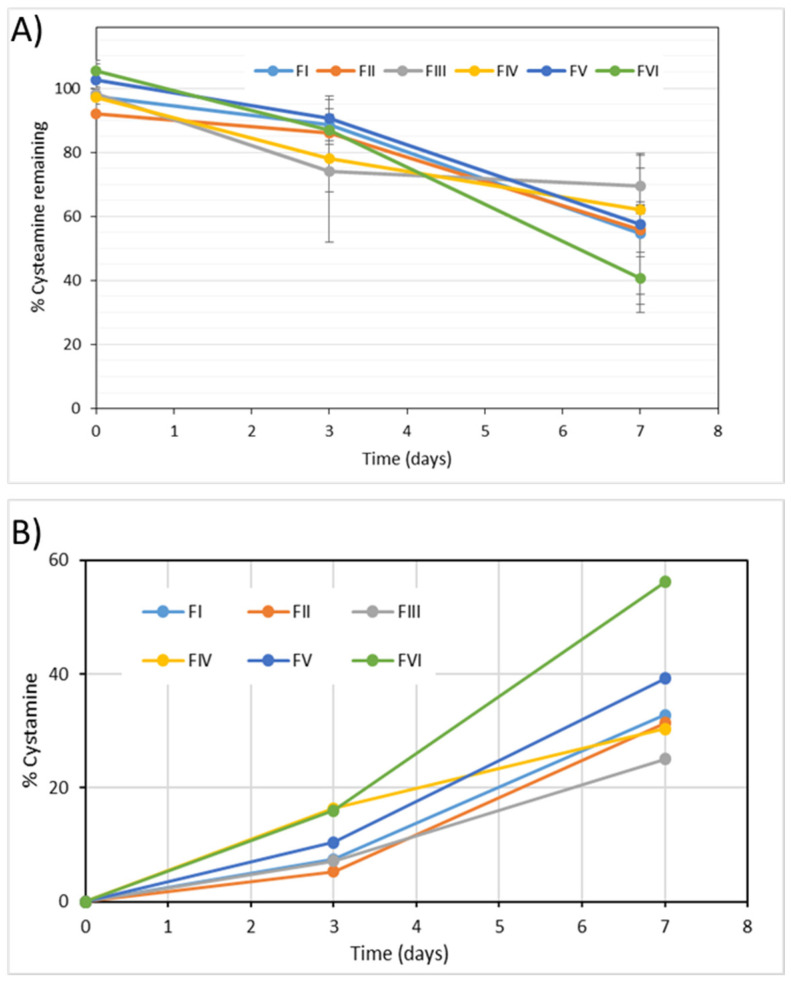
(**A**) Evolution of the percentage of cysteamine remaining in the different formulae during the in-use stability study. (**B**) Evolution of cystamine levels in the different formulae throughout the in-use stability study.

To determine the chemical stability of the samples simulating the conditions of use, the content of cysteamine and its main degradation product were quantified. In all the formulae a degradation of the drug was detected, which varied from 30% to 60% after 7 days’ use (Figure 7). As in the long-term study, although the differences were not significant, higher correlations were obtained assuming zero-order degradation kinetics. It is worth highlighting the high values of the degradation rate constants obtained in this study, which were significantly higher than those obtained in the long-term stability study (Table 4).

The most unstable formula was FVI, which was stored at room temperature, with a degradation rate constant statistically higher, and the most stable formula was FIII, which was stored in a refrigerator, saturated with nitrogen gas and lacked EDTA, although no statistically significant differences were detected between the degradation rate constants of the FI–FIV formulae.

Indeed, contrasting with the results of the long-term stability study, after 7 days’ use there were no major differences in the stability of the FI, FII, FIII and FIV formulations. After the first opening of the container in the in-use study, the effect of nitrogen gas on the stability of cysteamine was lost. Interestingly, the greater long-term stability of the FV formula compared to FII and FIV is not seen in the in-use study. On the contrary, the FV formula, with a simpler composition, had a significantly higher degradation rate constant.

Regarding the main degradation product, cystamine, in all formulae it was detected at a level greater than 20% after 7 days in use.

The highest percentage of cystamine was obtained with the FVI formula, stored at room temperature.

After 7 days of use, no significant differences were observed in terms of cystamine level among the FI, FII and FIV formulations, which was around 31%. The cystamine level in the FIII formula was slightly lower (25%), while in the FVI formula this value raised to 56%.

#### 3.3.4. Microbiological Stability

All formulations were stable from a microbiological point of view with respect to in-use stability conditions, since no contamination was detected in any of the evaluated conditions (blood agar, thioglycolate and Sabouraud agar).

## 4. Discussion

Cysteamine 0.55% eye drops are currently the only treatment available for ocular cystinosis, specifically cases of corneal cystine crystal deposition, with various studies having shown that it reduces photophobia and improves the quality of life of patients, while decreasing side effects, such as corneal erosions, scarring and neovascularization [18,31,32]. Although an EMA-approved pharmaceutical preparation is available [15], we still face the issue that it is not fully available in some European countries. Thus, off-license formulations prepared by hospital pharmacy services based on the formulations described in the literature are made available to patients [33]. In many cases, their expiration date does not guarantee their quality because it is not supported by rigorous stability studies, especially considering that cysteamine is a highly unstable molecule.

Considering the risk that the instability of this molecule may imply for patient health, along with the published studies that indicate the high lability of cysteamine in aqueous solutions [34,35,36], it was decided to evaluate the physical, chemical and microbiological stability of six formulations described in the bibliography over the course of their proposed long-term shelf life and in-use shelf life. To evaluate in-use stability, the conditions of use of these eye drops were simulated. It should be noted that the design of the work was focused on evaluating whether the stability of cysteamine in an aqueous medium is influenced by both the excipients of the formulation and the storage conditions.

All these studies have confirmed that cysteamine is a very unstable molecule in aqueous solution and that its almost exclusive route of degradation is oxidation, through which process cystamine is formed [34,37]. In fact, the decrease in cysteamine content in all the formulae studied both throughout the long-term stability study and in the in-use stability study, was accompanied by a proportional increase in the levels of its degradation product cystamine and the appearance of a characteristic hydrogen sulfide odor. Therefore, the presence of this characteristic odor could be used as a warning sign of drug degradation both by the patient and by the hospital pharmacy service. Oxidation reactions occur relatively rarely in pharmaceutical dosage forms as a main reaction, but when they do occur the formulation of stable preparations can be difficult.

On the one hand, one solution of cysteamine hydrochloride and EDTA in physiological serum was prepared to simulate its elaboration in a hospital pharmacy [27]. This is the simplest cysteamine eye drop formulation found in the literature. EDTA is a usual constituent of eye drop formulations. As a chelating agent, it prevents the catalysis of drug oxidation by metal ions potentially present in the solution, and also has antimicrobial activity [26]. This solution was packed in amber glass dropper bottles and stored under two different conditions: in a refrigerator (5 ± 3 °C) in the case of formulation FV and at room temperature (25 ± 0.5 °C) in the case of formulation FVI, in order to evaluate the effect of these storage conditions on the stability of the formulation.

As expected, it was observed that the higher the storage temperature, the greater the degradation of cysteamine in solution. The percentage of cysteamine degraded after one month of storage was, in both cases, very high—35% and 56% for FV and FVI, respectively, reflecting the high instability of cysteamine in aqueous solution even when stored in a refrigerator. Other authors have also described the effect of temperature on the chemical stability of cysteamine [18,36]. Specifically, Bozdağ et al. analyzed the stability of different formulations of cysteamine in solution with hydroxypropylmethyl cellulose (HPMC), EDTA and benzalkonium chloride, stored at room temperature and in a refrigerator at 4 °C. These researchers also observed significantly greater degradation in samples stored at higher temperatures [38]. Reda et al., who analyzed the stability of a 0.5% solution of cysteamine with benzalkonium chloride, concluded that the solution should be kept at −20 °C to guarantee its long-term stability, since they detected a 38% degradation of the active ingredient after one week stored in refrigerator [21].

The higher percentage of cysteamine degradation entails a proportional increase in the concentration of its main degradation product, and thus explains the earlier appearance of the unpleasant odor of hydrogen sulfide. The presence of cystamine in the formulations does not alter their pH values, their viscosity or their color because this degradation product does not present coloration.

The relationship between the storage temperature and the degradation rate of a chemical substance follows the Arrhenius equation. According to this equation, the ratio between the degradation rate constants (K) at two temperatures, in our case 25 °C and 5 °C, for a molecule with an average activation energy (E_a_) value in a solution of 83 KJ/mol (the E_a_ values for solutions usually oscillate between 40 and 125 KJ/mol) [39] would be 11 (Equation (1)).

(1) $$\frac{K_{25}}{K_{5}}=\frac{e^{-\frac{E_{a}}{R}\cdot \frac{1}{298}}}{e^{-\frac{E_{a}}{R}\cdot \frac{1}{278}}}$$

That is, the time necessary for a certain percentage of degradation of this molecule to occur at 5 °C is 11 times the time necessary for the same degradation to be detected at 25 °C [40].

However, from the cysteamine degradation data in the FV (5 °C) and FVI (25 °C) formulations obtained in the long-term stability study, the value calculated was 1.58. This indicates a low dependence of cysteamine stability and storage temperature, with an activation energy value calculated from this study data of 11.78 KJ/mol.

Regarding the in-use stability study, formulation VI stored at room temperature showed a degradation of the drug of 60% after 7 days and an intense unpleasant odour from the third day. Formulation V, stored in the refrigerator, showed a 43% degradation of the drug, and although these samples also had an unpleasant odour, it was not as intense. When calculating the ratio of the degradation rate constants at 25 °C and 5 °C, a value of 1.43 was obtained, similar to that obtained in the long-term stability study, confirming the low influence of the temperature of storage on cysteamine oxidation in these formulations.

In addition, during the in-use stability study, only a slight increase in pH values was observed after 7 days in use, this increase being higher in the case of formulation FVI (0.4 units) than for formulation FV (0.2 units). The rest of the parameters evaluated (viscosity, colour and microbiological stability) did not show modifications during the period tested.

All these results of the stability studies carried out on formulations FV and FVI make it possible to establish a low relationship between the degradation of cysteamine and the storage conditions, demonstrating that storage in a refrigerator significantly reduces the alteration of the aqueous solution of the drug but also showing that even when stored in the refrigerator, the instability of cysteamine in a simple saline solution with EDTA is too high to guarantee the efficacy of the formulation after a month of unopened storage and after a week of use. The small influence of the storage conditions on the oxidation of cysteamine in saline solution and EDTA makes it necessary to act at the formulation level in order to improve the stability of cysteamine in solution.

On the other hand, four formulae of cysteamine (FI–FIV) were elaborated from a 0.4% hyaluronic acid hydrogel in order to determine the influence of the constituents of the formulation on the stability of cysteamine in eye drops. Hyaluronic acid was used as a viscosifying agent to improve the permanence in the eye of the formulation [41]. Balanced salt solution (BSS) was used in all cases as a vehicle. BSS has a lower sodium chloride concentration than saline solution but incorporates other salts, such as potassium chloride, calcium chloride, magnesium chloride, sodium acetate and sodium citrate, presenting a composition more compatible with the ocular surface, which is why it is a very common vehicle in ophthalmic formulations. In the FI and FII formulations, but not in FIII and FIV, EDTA was also added as a chelating and preservative agent. Lastly, in FI and FIII solutions nitrogen gas was bubbled prior to packaging to remove dissolved oxygen. All the formulations were stored in a refrigerator at 5 ± 3 °C; therefore, their stability was compared with the FV formulation, previously analysed, made with physiological serum as a vehicle and 0.01% EDTA.

There were no significant variations in colour or pH values of any of the formulations, neither in the long-term stability study nor in the in-use study. Regarding viscosity, a significant decrease was detected in all formulations, more pronounced in FII and FIV in the long-term study (after one month it was reduced by 73% and 46%, respectively), and less pronounced in the case of the FIII formulation, with a 38% decrease at the end of the long-term study and 20% after 7 days in use. This significant decrease in viscosity is attributable to the alteration of hyaluronic acid. It has not been possible to relate this to the variation in cystamine levels in the formulae, although the saturation of the solutions with nitrogen seems to have reduced it. It also seems that the formulations without EDTA (FIII and FIV) showed a slightly lower decrease in viscosity compared to the formulations with EDTA (FI and FIV), an effect that was observed more clearly in the in-use stability study, where there was no overlap with the effect on viscosity of saturation of solutions with nitrogen. The decrease in the viscosity of the formulations would have as a consequence a reduction in the residence time of the drops in the corneal surface. This would make it necessary to increase the frequency of administration of the formulation, since, as previous studies have shown, the use of subtherapeutic doses of cysteamine, due to not following the administration guidelines for the eye drops, would result in a lack of therapeutic activity of the treatment [22,42].

Regarding chemical stability, the effect of EDTA on the stability of cysteamine in solution was evaluated. It is also the most frequent chelating agent used to reduce oxidation reactions catalyzed by metal ions. In our study, no statistically significant differences were detected between the degradation rate constants of cysteamine in the presence and in the absence of EDTA (FI vs. FIII and FII vs. FIV), neither in the long-term stability study nor in the in-use stability study, indicating that the degradation of cysteamine was not catalyzed by metal ions but through autocatalysis. This fact was also described by other authors who did not observe an improvement in the stability of cysteamine hydrogels due to the use of EDTA [26].

Since oxidation reactions occurring in aqueous solutions are a function of the concentration of oxygen dissolved in them, the best way to prevent them is to remove dissolved oxygen by nitrogen bubbling. To evaluate the effect of this strategy on the stability of cysteamine in solution, the FI and FIII formulations, which were saturated with nitrogen gas, were compared to the FII and FIV formulations, without nitrogen gas saturation.

In the case of the long-term stability study, the protective effect against the oxidation of nitrogen saturation of the solutions before packaging was evident. This protective effect is reflected in the degradation rate constant values, which were reduced by half or even a third in the formulae FI and FIII, saturated with nitrogen, compared to the formulae FII and FIV. This translates into degraded cysteamine values after one month of storage of 22% for formulations with nitrogen gas saturation and approximately 50% for the formulations without nitrogen gas saturation.

The stabilizing effect of nitrogen gas on cysteamine in solution was lost when the containers were opened for the first time in the in-use stability study. Thus, the differences between formulations saturated with nitrogen gas and formulations without nitrogen saturation were less evident, with not statistically significant differences between the degradation rate constant values (FI vs. FII and FIII vs. FIV).

As previously mentioned, another effect of the removal of oxygen from cysteamine solutions was a lower degradation of hyaluronic acid, which was manifested in a decrease in the viscosity of the samples of less than 40% in formulae FI and FIII compared to the decrease in viscosity of 73% and 46% in the FII and FIV formulations, respectively. This effect was also not observed in the in-use stability study.

All these results show that nitrogen gas saturation was key for reducing the instability of the cysteamine solutions during storage. However, it is important to highlight two facts. First, the difficulty of applying this resource at the hospital pharmacy level, since a source of gaseous nitrogen and a system for bubbling it in the solutions (which have to be kept in sterile conditions until closure of the bottles) is needed. Not all hospital pharmacy services have the necessary equipment to carry out this process. Second, despite the reduction in oxidation observed with this strategy, none of the analysed formulae showed a cysteamine degradation less than 20% nor a decrease of viscosity less than 35% after one month of storage in the refrigerator. They also did not show a decrease in cysteamine content of less than 30% and in viscosity of less than 20% after 7 days of use.

Regarding the formulae not saturated with nitrogen, it is worth noting the greater long-term stability of the FV formulation, made only with EDTA and saline as a vehicle, compared to the FII formulation, with EDTA, hyaluronic acid and BSS as a vehicle. However, in the case of the in-use stability study, the situation was reversed, probably because the lower viscosity of the FV formula favoured the interposition of the new supply of oxygen that came into contact with the solution each time the container was opened. Indeed, although it is generally assumed that the equilibrium between gaseous and dissolved oxygen is instantaneous, when the system is static or is subjected to low agitation it can be diffusion-controlled [43].

Finally, it is interesting to note that the oxidation of cysteamine in the in-use stability study of each of the elaborated formulae was much faster than that detected in the long-term study, the samples, in both cases, kept under the same storage conditions (5 °C). Indeed, in the long-term study, the samples behaved as a closed system, with an equilibrium between dissolved oxygen, responsible for the reaction with cysteamine, and the gaseous oxygen trapped in the container, so that as the oxidation reaction progressed the available oxygen was consumed and the reaction slowed down. This did not occur in the in-use stability study, in which the samples behaved as an open system, in which the oxygen consumption by the degradation reaction was compensated with a new supply of atmospheric oxygen each time the container was opened.

In general, it should be noted that all the formulae studied showed high instability at the end of the long-term and in-use stability studies. Specifically, at the end of the long-term stability study, cysteamine content values were approximately 78% of the labelled, in the case of FI and FIII formulations, and less than 45% in the case of the FVI formulation. The literature indicates a shelf life of 30 days in the case of hydrogels (FI–FIV) [26]. Surprisingly, cysteamine solutions prepared only with physiological saline and EDTA (FV and FVI) are assigned a shelf life of up to 4–6 months [27].

Likewise, at the end of the in-use stability study, the cysteamine content in the formulations ranged between 69% in the case of the FIII formulation and less than 41% in the FVI formulation. In all cases, these values are very far from the limits established by the FDA to establish the period of use once opened (defined by the FDA guidelines as “more than 90% of drug”) [34]. The in-use shelf life designated in the literature for this type of formulation is 7 days for all formulae except the FV formulation, for which some references assign an in-use shelf life of 15 days. We extended the in-use stability study for the FV formula up to 15 days, obtaining a cysteamine content of 1% at the end of this period, making plain the risk of therapeutic failure that these data suggest.

## 5. Conclusions

After the long-term and in-use stability studies carried out on six compounded formulations of cysteamine eye drops, we can conclude that cysteamine is a very unstable molecule in aqueous solution, undergoing a rapid oxidation even when preparations are stored in the refrigerator. Its main degradation factor is the presence of oxygen, which, when reacting with cysteamine, gives rise to cystamine and the appearance of a characteristic hydrogen sulfide odor that serve as an indicator of the alteration of the preparation.

Among all the variables studied to improve the stability of cystamine in solution, the one that provided the best results was the saturation of the solutions with nitrogen. Despite this, in all cases the degradation of cysteamine was greater than 20% in a long-term stability study with a duration of one month (the shelf life proposed in the bibliography); and more than 35% in a 7-day in-use stability study (the in-use shelf life proposed in the bibliography). This high degradation of cysteamine could seriously compromise the clinical efficacy of these formulations.

These results signal the need to search for other formulations of cysteamine that will ensure the stability of the drug throughout its shelf life and during use and the obligation to carry out rigorous stability studies that will guarantee the validity of all formulations of cysteamine eye drops that are used in the clinic.

## Figures and Tables

**Table 1:** Composition and storage specifications of the developed formulae.

	Composition Per Vial					
	F I	F II	F III	F IV	F V	F VI
Cysteamine (%)	0.55	0.55	0.55	0.55	0.55	0.55
Hyaluronic Acid (%)	0.4	0.4	0.4	0.4	---	---
EDTA (%)	0.01	0.01	---	---	0.01	0.01
BSS (mL) q.s.	10	10	10	10		
Physiological saline solution (mL) q.s.	---	---	---	---	10	10
Vial	Sterile class I 15 mL amber glass dropper bottle					
Saturation with N2	Yes	---	Yes	---	---	---
Storage conditions	Refrigerator (2–8 °C)					Room temperature (25 °C)

**Table 2:** Gradient HPLC method for quantification of cysteamine and cystamine. The mobile phase A was an aqueous sodium heptane sulfonate solution (pH 2.5) and the mobile phase B was acetonitrile.

Time (min)	Mobile Phase A (%)	Mobile Phase B (%)
0	80	20
1.0	80	20
16.0	35	65
16.1	10	90
19.0	10	90
19.5	80	20
24.0	80	20

**Table 3:** Degradation rate constants (K) obtained from the data generated in the long-term stability study. S_k_: standard deviation of K; r: correlation coefficient of percentage of drug remaining vs. storage time.

	K (%/day)	S_k_ (%/day)	r
FI	0.5343	0.1329	−0.7859
FII	1.5866	0.0811	−0.9872
FIII	0.7328	0.0985	−0.9274
FIV	1.493	0.1191	−0.9696
FV	1.2095	0.1132	−0.9589
FVI	1.906	0.2069	−0.9458

**Table 4:** Degradation rate constants (K) obtained from data of the in-use stability study. S_k_: standard deviation of K; r: correlation coefficient of percentage of drug remaining vs. in-use time.

	K (%/day)	S_k_ (%/day)	r
FI	5.2301	0.9119	−0.9573
FII	5.3082	1.1188	−0.9215
FIII	4.1976	1.6782	−0.781
FIV	4.9523	0.899	−0.94
FV	6.5433	1.7833	−0.878
FVI	9.3489	1.0367	−0.9763

## Data Availability

The data presented in this study are openly available in Research gate at https://doi.org/10.13140/RG.2.2.16027.59682.
